# Interpreting growth hormone and IGF-I results using modern assays and reference ranges for the monitoring of treatment effectiveness in acromegaly

**DOI:** 10.3389/fendo.2023.1266339

**Published:** 2023-10-25

**Authors:** David R. Clemmons, Martin Bidlingmaier

**Affiliations:** ^1^Department of Medicine, University of North Carolina at Chapel Hill, Chapel Hill, NC, United States; ^2^Neuroendocrine Unit, Medizinische Klinik und Poliklinik IV, Klinikum der Universität München, Munich, Germany

**Keywords:** biochemical markers, growth hormone, IGF-I, therapy, treatment response, acromegaly

## Abstract

Standard treatment for acromegaly focuses on the achievement of target absolute levels of growth hormone (GH) and insulin-like growth factor (IGF-I). The appropriateness of these targets when measured using modern assay methods is not well defined. This paper reviews biochemical status assessed using methods available at the time and associated clinical outcomes. GH measurements were shown to provide an indication of changes in tumor size, and failure of GH suppression after glucose stimulation is associated with tumor recurrence. IGF-I levels were more closely associated with changes in symptoms and signs. Reduced GH and IGF-I concentrations were shown to be associated with increased longevity, although the degree of increase has only been analyzed for GH. Lowering of GH and IGF-I has consistently been associated with improved outcomes; however, absolute levels reported in previous studies were based on results from methods and reference ranges that are now obsolete. Applying previously described absolute thresholds as targets (e.g. “normal” IGF-I level) when using current methods is best applied to those with active acromegaly symptoms who could benefit from further lowering of biochemical markers. In asymptomatic individuals with mild IGF-I or GH elevations, targeting biochemical “normalization” would result in the need for combination pharmacotherapy in many patients without proven benefit. Measurement of both GH and IGF-I remains an essential component of diagnosis and monitoring the effectiveness of treatment in acromegaly; however, treatment goals based only on previously identified absolute thresholds are not appropriate without taking into account the assay and reference ranges being employed. Treatment goals should be individualized considering biochemical improvement from an untreated baseline, symptoms of disease, risks, burdens and costs of complex treatment regimens, comorbidities, and quality of life.

## 1 Background

Acromegaly is a disease with a slow and insidious beginning in which the symptoms and signs of excess growth hormone (GH) and insulin-like growth factor (IGF-I) take several years to become overtly manifest. Consequently, the disease often goes undetected for periods of 8 to 10 years following onset, at which time multiple comorbidities and a large pituitary tumor that is difficult to resect may exist (1). Early diagnosis would obviate some of these problems, leading to a higher rate of surgical cure and the development of fewer and less severe comorbidities. However, in spite of marked improvements in the tools for establishing a definitive diagnosis, including GH and IGF-I assays as well as magnetic resonance imaging, patients routinely present with symptoms and signs that have progressed to a relatively advanced state (1). Therefore, at present, while surgery is the treatment of first choice, physicians and patients are often left with difficult choices following non-curative debulking of the pituitary tumor. Developments in assay methodology have improved the utility of GH and IGF-I to assess disease activity. However, making therapeutic decisions with the results has been complicated by the need to conduct long-term studies to determine the efficacy of various therapies and to understand the relationship between GH and IGF-I levels and long-term outcomes. The goal is complicated by the ongoing evolution of assay methodology and normal reference ranges (2). The major purpose of this review is to assess the published evidence and treatment guidelines (which are often based on consensus reports and not prospective studies) regarding GH and IGF-I assays and their changes over time and provide recommendations for how this knowledge is best applied to the management of this complex disease.

## 2 Physiological control of GH and IGF-I

GH is stimulated by the hypothalamic releasing factors growth hormone–releasing hormone and ghrelin and inhibited by the hypothalamic peptide somatostatin (3). GH is secreted in a pulsatile manner, and its level fluctuates in response to food, stress, exercise, and sleep. GH secretion is also influenced by body mass index (BMI) and estrogen status. Therefore, random GH concentrations cannot be used to distinguish between normal and pathological GH levels. IGF-I secretion, however, is much more consistent throughout the day because it circulates bound to binding proteins, which extend its half-life to 16 hours, and also because its concentrations, while GH-dependent, do not fluctuate acutely. IGF-I synthesis is inhibited by reduced nutrient intake and liver disease.

Acromegaly is caused by a GH-secreting adenoma of the pituitary that appears most commonly in patients between the ages of 35 and 45. The initial symptoms and signs include headaches, swelling of the hands and feet, subcutaneous tissue thickening, and acral bone growth resulting in ring and shoe size changes, increased sweating, and arthralgias. Because these changes are slow and insidious, they frequently go unrecognized by the patient’s immediate family members and the primary care provider. Many times, the diagnosis is made when the patient changes healthcare providers or is referred to a specialist who has not previously had an encounter with the patient. Because this is a rare disease (estimated prevalence 33 cases per million) (4), the patient and immediate family members are often unaware of its existence. GH and IGF-I are tests that are used infrequently by primary care providers and, therefore, they are not generally used to screen patients with symptoms that in isolation may be perceived as non-specific. These factors all contribute to the delay in diagnosis, but once the diagnosis is suspected it is rapidly confirmed by measurement of GH and IGF-I.

## 3 GH and IGF-I in the diagnosis of acromegaly

The use of GH and IGF-I measurements to establish the diagnosis has been supported by multiple studies (3, 5, 6). Importantly, GH and IGF-I provide complementary but different information, with GH being a tumor marker and IGF-I being the better measure of the symptomatic and metabolic consequences of acromegaly (7, 8). IGF-I is the preferred diagnostic test since the presence of acromegaly in the context of a normal age-related serum IGF-I is exceedingly unusual. Furthermore, unlike GH, random IGF-I measurements are not hindered by food intake, time of day, or in most cases concomitant medications. In the great majority of individuals presenting with signs and symptoms suggestive of acromegaly, a random serum IGF-I will be unequivocally elevated and no further biochemical confirmation is required. In patients in whom the diagnosis remains in doubt, an oral glucose tolerance test should be undertaken. In healthy individuals, acute elevation of plasma glucose typically suppresses GH to a level <0.3 ng/mL when the measurement is performed using a sensitive assay (9).

## 4 GH & IGF-I as markers of disease activity

Basal fasting GH values have been shown to provide a good index of surgical prognosis (7). IGF-I values aid in determining disease severity at the time of diagnosis (8). Specifically, IGF-I levels correlate with the degree of soft tissue enlargement, as well as the severity of insulin resistance, as determined by measurement of blood glucose and fasting insulin values (10). Additionally, there is an association with the degree of increase in IGF-I and presence of other comorbidities such as sleep apnea, cardiomyopathy, hypertension, and arthritis (11).

## 5 Monitoring the response to surgery and or radiotherapy

Postoperative evaluation should include not only screening for pituitary hormone deficiencies but also measurement of random GH values as well as IGF-I. Some studies have strongly supported measuring GH after glucose suppression because failure to suppress to normal has been shown to be associated with a higher rate of tumor recurrence (12). GH measured relatively early after surgery often has good predictive value for surgical cure; the optimum time for determining IGF-I has been a subject of great debate, but in general this is believed to be between 6 weeks and 3 months following surgical resection (13). Sometimes there is transient damage to the GH-producing cells in the tumor, which can lead to a false conclusion that the resection was total. Additionally, IGF-I has been shown to decrease slowly in some patients after surgery; therefore, determining that the patient has reached a basal value by utilizing more than one IGF-I determination will help to establish the degree of improvement that has occurred with surgery. Patients who have a stable IGF-I within or near the normal range will benefit from repeating the GH suppression test to determine if the disease is completely resolved. The GH suppression test is preferred because GH is secreted in a pulsatile manner (14), and if suppression is normal, this suggests surgery has likely returned GH secretion to a normal physiologic state. Patients who fail to suppress GH after administration of oral glucose or who still have postoperative elevation of fasting GH often have a higher recurrence rate; therefore, these patients should be followed closely, even if they have a normal or near-normal IGF-I level (1.0× to 1.3× the upper limit of normal [ULN]) postoperatively (12). Additionally, the presence of pituitary hormone deficits (e.g. hypothyroidism) can influence IGF-I and GH values; therefore, other hormone levels (e.g. T4) should be quantified and, if indicated, replacement therapy should be undertaken prior to measurement of GH and IGF-I (15).

Following definitive determination of the stable GH and IGF-I values postoperatively, a decision should be made regarding the need for additional therapy. Prior to the advent of treatment with somatostatin analogs and dopaminergic agonists, the only additional modality available was radiation therapy. However, multiple long-term outcome studies have shown that in addition to inducing hypopituitarism, which develops in most patients if followed for sufficient duration (16), radiation therapy is associated with an increased risk of stroke (17). Several studies that analyzed treatment outcomes have shown that the relative risk of mortality is significantly higher in patients who received radiotherapy when compared to patients who did not receive conventional radiotherapy (17–20). It should be noted that these analyses do not include long-term follow-up of patients who were treated with newer radio-surgical techniques that have been introduced relatively recently (21). An additional problem with radiotherapy is the slow rate of decrease in GH and IGF-I secretion, which correlates with slow changes in clinical disease activity. Therefore, at present, the general recommendation is to reserve radiotherapy for patients with definitive evidence of growing pituitary tumors or tumors positioned where they may induce damage to neurologic structures, primarily the optic chiasm (22). The other potential use of radiotherapy is in patients who have failed to respond adequately to drug therapy, or in those for whom pharmacotherapy is not possible (22). In conclusion, the general outcome after surgery will be in one of three categories, based on assessment of GH and IGF-I: (I) patients are cured surgically with normal IGF-I and suppressible GH; (II) patients have normal or near-normal IGF-I (1.0× to 1.3× ULN) and minimally elevated basal GH or non-suppressible GH but are asymptomatic, in which case they should be followed closely but do not require pharmacotherapy (6, 12); or (III) it has been determined definitively that patients have a need for further therapy (pharmacotherapy, radiation therapy, or repeat surgery). This decision should be based on the presence of symptoms or signs (**Table 1**) (23–25), progression of comorbidities, and increasing tumor size, as well as GH and IGF-I levels.

**Table 1:** Clinical manifestations of acromegaly (23).

Mass effects of tumor	Headache, visual impairment, hyperprolactinemia, pituitary stalk section, hypopituitarism, hypothyroidism, hypogonadism, hypocortisolism
Systemic effects of excess GH/IGF-I	Soft tissue and skin changes, acral enlargement, increased skin thickness and soft tissue hypertrophy, increased sweating, skin tags, and acanthosis nigricans
Cardiovascular features	Hypertrophy, congestive heart failure, coronary disease, arrhythmias, hypertension, cardiomyopathy
Metabolic features	Impaired glucose metabolism, diabetes, insulin resistance
Respiratory features	Macroglossia, upper airway obstruction, sleep apnea, ventilator dysfunction
Bone and joint features	Increased articular cartilage thickness, arthroplasty/osteoarthritis, carpal tunnel syndrome, vertebral fractures
Other endocrine consequences	Goiter, hypercalciuria, menstrual abnormalities

GH, growth hormone; IGF-I, insulin-like growth factor. Reprinted with permission from Cordero and Barkan, *Rev Endocr Metab Disord* 2008;9(1):13-19, © Springer Nature, with additional data from Abreu et al. and Madeira et al. (22–24).

## 6 Medical therapy

Once a decision has been made to begin medical therapy there are three general options available. Two classes of medications work directly on the pituitary tumor to suppress GH secretion: dopaminergic agonists (e.g. cabergoline) and somatostatin receptor ligands (e.g. octreotide, lanreotide, pasireotide), while pegvisomant as a GH receptor antagonist lowers IGF-I by blocking GH action. Treatment with somatostatin analogs can be associated with a modest reduction in tumor size, whereas the GH receptor antagonist has no effect on tumor size. In general, dopaminergic agonists and somatostatin analogs are used as first-line therapy because of safety and cost. Cabergoline may be effective in patients with IGF-I values <50% above the ULN, but for patients with IGF-I values higher than that, the probability of normalizing IGF-I is low (26). Therefore, these patients are usually started on somatostatin analogs. Pasireotide is a somatostatin receptor subtype 2 (SST2) agonist, but it also has potent somatostatin receptor subtype 5 (SST5) agonist activity that accounts for its ability to suppress insulin secretion (27). In a head-to-head trial, pasireotide normalized IGF-I in 31.3% of patients, whereas octreotide normalized it in 19.7% (28). The patient population reflected typical post-surgical patients, as 58% were naive to medical therapy and the mean IGF-I was 3.1× ULN. Comparison of lanreotide and octreotide long acting formulations has shown they are equipotent in most studies (29, 30), except one that found a better response to octreotide (31). Pegvisomant has a higher rate of IGF-I normalization (67-95%) but requires daily injections and is often the most expensive option (32, 33). If single-agent therapy is unsuccessful, often the combination of dopaminergic agonist and somatostatin analog (34) or somatostatin analog plus pegvisomant is used (35). Somatostatin analogs may be associated with increased or decreased glucose levels (36). This contrasts with the GH receptor antagonist, which may provide additional reduction in insulin resistance (37). Therefore, the factors that are taken into account in choosing a particular medication include the size and location of the residual tumor, the degree of residual abnormality of IGF-I secretion, and the presence or absence of diabetes. In patients with relatively high IGF-I values but residual large tumors, the decision is often made to begin with somatostatin analogs in the hope of inducing some tumor shrinkage or at least preventing further tumor enlargement. Patients with intractable headaches may also benefit from somatostatin analogs. Growth hormone receptor antagonist therapy may benefit patients with diabetes mellitus or glucose intolerance (38).

In evaluating the response to medical therapy, it is important to allow sufficient time for drugs to reach optimal plasma concentration and achieve their maximal target effects. In general, dopaminergic agonist efficacy can be assessed relatively rapidly after four weeks of therapy and a decision can be made as to whether to continue the agent, add an additional agent, or discontinue the agent and switch to a new form of therapy. In contrast, long-acting somatostatin analog injections usually require three months per dose to assess efficacy; therefore, titration to the maximum dose may be prolonged. For example, because there are 4 approved doses of octreotide LAR, this process could render the patient suboptimally controlled for 1 year while a full titration is performed. It is important to remember that assessment of the degree of response should include a comprehensive evaluation of multiple factors, and that simply relying on the response of GH and IGF-I is often inadequate. Consideration should be given to changes in symptoms, signs, and comorbidities (**Table 1**) (23–25, 39, 40). Symptoms that are often closely correlated with biochemical improvement include decreases in sweating, headaches, soft tissue swelling, and arthralgias (41). The most helpful changes in signs of acromegaly, as indicators of treatment response, are decreased ring size and skin thickness. Some improvements are drug-specific; for example, a change in pituitary tumor size in response to somatostatin analogs may be reflected by improvement in visual acuity or decreased headaches. There was concern initially that pegvisomant treatment might result in tumor enlargement but that has not been confirmed in several studies (42). Similarly, improvement in insulin sensitivity in response to pegvisomant may result in decreased need for diabetes medications. However, changes in comorbidities often progress slowly and may require additional therapeutic modalities that are specifically directed at improving the comorbidity (24). Specifically, optimal control of blood pressure and blood glucose will usually require additional therapies, as will improvements in osteoarthritis, sleep apnea, and cardiomyopathy.

## 7 Biochemical monitoring

Regarding biochemical monitoring, both IGF-I and GH levels should be measured since they yield qualitatively different types of information. IGF-I correlates most closely with symptomatic improvement and changes in signs (39, 43), whereas GH directly measures tumor output (44). However, it should be noted that studies often do not report changes in both IGF-I and GH and correlate them with a quantitative measure of improvement in symptoms or signs; therefore, few studies exist wherein these direct comparisons have been made (10, 24, 39, 41). Nevertheless, those studies tend to favor the utilization of IGF-I to monitor changes in symptoms and signs, whereas changes in tumor size and tumor recurrence are most often related to changes in GH secretion (7, 45, 46). In situations for which GH and IGF-I are not reliable indicators of a change in comorbidities, monitoring should include direct testing of the consequences of the specific comorbidity (e.g. formal sleep studies, pulmonary function testing, echocardiography). Assuming the maximal therapeutic dose has been achieved using a single therapeutic agent, for patients who remain actively symptomatic and have significant elevations of GH or IGF-I, clinicians must decide whether to add a specific treatment modality or to discontinue the current medication and initiate therapy with a single alternative agent. The same variables that were assessed in the initial postsurgical evaluation should be utilized to make this decision (e.g. persistence of symptoms and signs, presence or absence of comorbidities, location and size of residual tumor, presence of specific comorbidities such as diabetes). Additionally, the degree of biochemical response that occurred with the initial therapy should be considered. For example, if initiation of therapy with cabergoline has resulted in minimal improvement (e.g. a 10-20% reduction in GH and/or IGF-I), often it will be more efficacious to change to an alternative class of medication. In practice this usually means that somatostatin analogs should be introduced if response to cabergoline is inadequate. In contrast, if the response to somatostatin analogs has been established as inadequate, changing to pegvisomant as a sole therapy is a viable option. It is important to remember that this decision should not be based on a single biochemical test but rather take into account each of the variables mentioned previously (1, 4, 6, 22, 47).

## 8 The evolution of changes in the use of biochemical testing to monitor the response to pharmacotherapy

In the 1960s, the introduction of radioimmunoassay of GH provided a major tool to assess the biochemical activity of the disease state following treatment. With the advent of transsphenoidal surgery in the early 1970s, this test was utilized to assess residual disease activity following adenectomy. The initial criteria for surgical cure were random GH values <5 ng/mL; however, this was followed in the late 1990s with the recommendation that surgical cure was more likely if the GH value was <2.0 ng/mL (48). With regard to monitoring the response to therapy with GH measurements, it has been vigorously debated as to whether glucose-suppressed GH, a random single GH, or multiple random GH measurements (termed a “day curve”) have the greatest value (49–52). Some studies have shown a high degree of correlation between random GH measurements and glucose-suppressed GH or day curve measurements, and some have not (50–54). Therefore, it remains difficult to determine which measurement is the most reliable for predicting a satisfactory response to treatment. Certain drugs, such as somatostatin analogs, interfere with GH suppression, suggesting that GH suppression should not be used to monitor patients being treated with these agents (55). In the past, another disadvantage of using glucose-suppressed GH values was that age- and gender-adjusted normative ranges had not been determined (56). However, these have recently been published (57). Because pegvisomant is a GH receptor antagonist, it results in elevation in GH; therefore, IGF-I is the only acceptable biochemical test that can be used to monitor these patients (41). Taking these factors into consideration, in non–pegvisomant-treated patients, random GH measurements are generally preferred, since most of these patients will be on somatostatin analogs. The ability to perform repetitive measurements over a period of time (day curve) may be superior to a single GH measurement; however, this may not be possible in the outpatient setting in some clinics (51). The exact GH value used to determine whether therapy is adequate is dependent upon the type of assay and the specific reference ranges used for that assay (see section on GH and IGF-I assays). When basal GH values are being measured, the sample should be obtained fasting.

IGF-I values also require age- and gender-adjusted normative ranges (58). These can vary greatly among reference laboratories (59, 60). The types of IGF-I assays vary widely; therefore, it is always preferable to continue to use the same assay for an individual patient when assessing the response to therapy. In general, it is accepted that an IGF-I within the normal range defines an optimal response to surgery (1). When the results of GH and IGF-I measurements confirm that control of the disease has been achieved, the patient can be safely maintained on that therapy but should be monitored at 6-month intervals to confirm that control is maintained. In patients in whom both GH and IGF-I measurements confirm lack of control and who are symptomatic or show progression of comorbidities, the next step would be to either change therapy or treat with a combination of agents. As noted previously, factors such as the degree of biochemical response to the initial therapy, the presence of diabetes, and tumor size should be taken into consideration.

**Figure 1:**
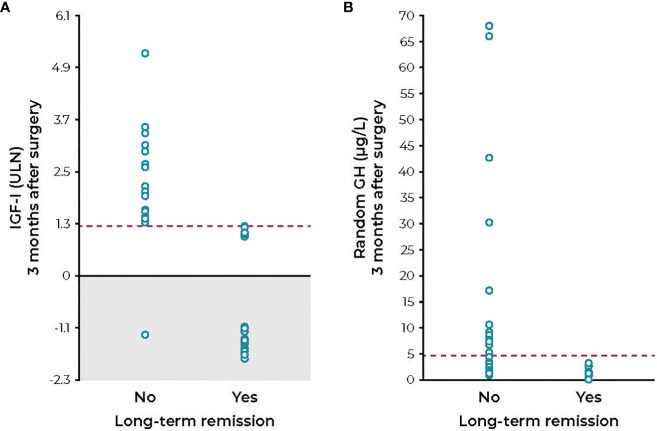
IGF-I levels **(A)** and random GH levels **(B)** measured 3 months after transsphenoidal surgery in acromegaly patients who progressed with or without long-term remission. GH, growth hormone; IGF-I, insulin-like growth factor; ULN, upper limit of normal. Figure reprinted with permission from Cunha et al. *Endocrine* 2020;68(1):182-191, © Springer Nature (69).

A major problem for clinicians has been determining the approach to therapy when the IGF-I or GH value is increased but near the normal range and the patient has minimal or no symptoms or comorbidity progression. Studies that have used arbitrary cutoffs between 1.2× and 1.3× ULN for IGF-I have greater sensitivity for predicting symptomatic improvement or stability; whereas, if this cutoff is lowered to strictly within the normal range, sensitivity is reduced (61–66). Freda has studied such patients in detail and has concluded that there is insufficient evidence indicating that GH levels need to be suppressed into the normal range in asymptomatic patients (39, 67). She also concluded that when the IGF-I level is near normal it remains controversial as to whether a patient without symptoms requires treatment (67). Furthermore, because of changing reference ranges and assay methods, as well as intrinsic biological variability in IGF-I secretion, values can clearly be as high as 1.3× ULN in patients who are asymptomatic (64, 66–69). Patients have been reported who had abnormal values after surgery (between 1.0× and 1.3× ULN) and attained normal values after one year of follow-up while receiving no therapy (**Figure 1**) (66, 69). Additional follow-up for four years showed no tumor or symptomatic recurrence in these patients. In contrast, 25 patients with IGF-I values >1.3× ULN who were followed in the same study had persistent tumors, were symptomatic, and required medical therapy. The explanation for the spontaneous decrease in IGF-I has not been forthcoming; however, similar cases have been described and reported (47).

Several studies have shown there may be significant improvements in specific comorbidities such as diabetes in patients who have a substantial reduction in IGF-I, even though the IGF-I value remains above the normal range (39, 66, 68–72). In patients with active symptoms or comorbidity progression, the decision is more straightforward and, generally, treatment is initiated. However, in patients with mild elevation of IGF-I without symptoms or comorbidities, there is no consensus as to whether they should receive drug therapy if IGF-I values are between 1.0× and 1.3× ULN.

## 9 The problem of discrepant IGF-I and GH values

In the early 1980s, radioimmunoassay of IGF-I became widely available and, therefore, studies could compare the prognostic value of an IGF-I measurement after transsphenoidal adenectomy as compared to measurement of random GH, GH day curve, or GH after glucose suppression. Preoperative IGF-I values were also analyzed for their ability to predict surgical improvement. Most studies have shown that a random GH value of >10 ng/mL post-surgery has superior predictive value of the likelihood of surgical cure (73, 74). Most large series assessing the response to transsphenoidal surgery have shown that IGF-I and GH responses are discordant in 5.4-39.5% of patients (74, 75). Similar proportions of discordant values have also been reported following medical therapy (55, 71, 76) and higher variations following radiotherapy (52). Discordant values can occur in either direction: IGF-I can be significantly elevated with a normal basal or glucose-suppressed GH; conversely, IGF-I can be normal and GH elevated (**Table 2**) (67). It is important to remember there is a logarithmic relationship between increasing GH and an increase in IGF-I; therefore, the degree of abnormality in GH may appear to be greater (77). One explanation for an elevated GH with a normal IGF-I has been the presence of estrogen, either due to the use of estrogen medications (oral estrogen medication has a greater effect compared with transdermal delivery of estrogen) or in women who retain intact ovarian function (71). In these cases, estrogen acts as a GH antagonist and there is resistance to endogenous GH, resulting in a normal IGF-I and a slightly elevated GH (78). Patients not taking estrogen—for whom GH remains elevated after surgery, but IGF-I is normal—are at increased risk for tumor recurrence. As mentioned previously, GH represents a direct measurement of tumor output and, therefore, it is a more accurate index of the likelihood of a recurrence (39).

**Table 2:** Causes of discrepant IGF-I and GH values in patients with acromegaly (64).

	“Abnormal” GH suppression with a normal IGF-I level	“Normal” GH suppression with an elevated IGF-I level
**Patients in remission**	• Dysregulation of GH secretion – Disruption of the neural or anatomic networks of GH regulation – Mild or early GH excess? • Causes of abnormal GH suppression other than acromegaly – Chronic renal insufficiency – Liver failure – Active hepatitis – Anorexia nervosa – Malnutrition – Hyperthyroidism – Diabetes mellitus – Adolescence • Cut-off for GH suppression inappropriately low for the GH assay used	• Falsely elevated IGF-I – Adolescence – Pregnancy – Hyperthyroidism (mild elevation) – IGF-I assay problems • Early postoperative period • By definition, 2.5% of normal people have an elevated IGF-I
**Patients with active disease**	• Lowering of the serum IGF-I level – Nutrient deprivation, malnutrition – Anorexia nervosa – Liver disease – Hypothyroidism – Poorly controlled insulin- dependent diabetes mellitus – Oral estrogen use • Inaccurate IGF-I normal range (upper limit too high)	• Cutoff for GH suppression too high for the GH assay used • Easily suppressible early or mild active acromegaly

GH, growth hormone; IGF-I, insulin-like growth factor. Adapted with permission from Freda *Clin Endocrinol (Oxf)* 2009;71(2):166-170, © Blackwell Publishing Ltd. (64).

Several reports have described patients in whom GH values are relatively low but IGF-I is significantly increased and greater than >1.3× ULN. Generally, these patients are symptomatic and may have comorbidities such as diabetes (43). One study showed patients with high IGF-I and normal GH were more likely to have elevated blood glucose and hypertension (71). One explanation is that IGF-I reflects 24-hour GH secretion, and that patients who secrete a low level of GH throughout a 24-hour interval may have low GH values at a single point in time that do not accurately reflect their 24-hour GH secretion rate (39, 43). Another reason proposed for this discrepancy is failure to measure the 20 kDa form of GH, which is biologically active and stimulates IGF-I. Conventional radioimmunoassays often detect 20 kDa GH (79); however, newer assays using monoclonal antibodies that are prepared against 22 kDa GH may have minimal cross-reactivity with 20 kDa (80, 81). Since the 20 kDa form of GH is biologically active, this could result in an elevated IGF-I, whereas the measurement of 22 kDa GH may appear normal.

During treatment with somatostatin analogs, discrepant values between GH and IGF-I have been reported in 13.7-35.4% of cases (55, 71, 76). In a study using a Belgian database, 26 of 99 patients (26.3%) showed discordance, with a high IGF-I and normal random GH (82). Following extensive evaluation to exclude patients with confounding factors such as estrogen dosing or radiation therapy, only 15% of the values were discordant, and the authors commented that discordance, when present, was generally mild. Furthermore, no adverse outcomes were observed in patients with borderline elevated IGF-I levels, as assessed by the presence of diabetes or hypertension during a follow-up period of six to nine years (83). In this study, the cutoff for normal GH was 2 ng/mL. Other causes of discordance that have been proposed include inaccurate GH or IGF-I measurements, disturbed GH pulsatile secretion, tonic GH secretion, differences in target tissue sensitivity, the method of IGF-I assay, and GH receptor polymorphisms (74, 84). Some studies have shown that treatment with somatostatin analogs is more likely to result in discordant values, and in one study 43% of patients showed discordance (85, 86). Importantly, however, IGF-I and GH, while discrepant, were both significantly lower in controlled compared with uncontrolled patients (86). In the discrepant group, IGF-I was 26% above ULN in controlled asymptomatic patients versus 88% above ULN in the discrepant, uncontrolled, symptomatic patients. Similarly, the mean GH value was 0.7 ng/mL in the controlled discrepant patients. By contrast, a large Italian study showed lower divergent values. In a study of 279 oral glucose tolerance tests in 93 patients treated either with dopaminergic or somatostatin agonists, 12% of GH values after oral glucose tolerance test suppression were discordant with baseline, and the basal value was discordant between GH and IGF-I in 30% (54). Treatment with either dopaminergic agonists or somatostatin analogs resulted in a similar percentage of discordant values for each treatment (54), suggesting that discrepancies are not medication-dependent.

## 10 Relationship between GH or IGF-I and clinical outcomes in patients with acromegaly

### 10.1 Symptoms

**Figure 2:**
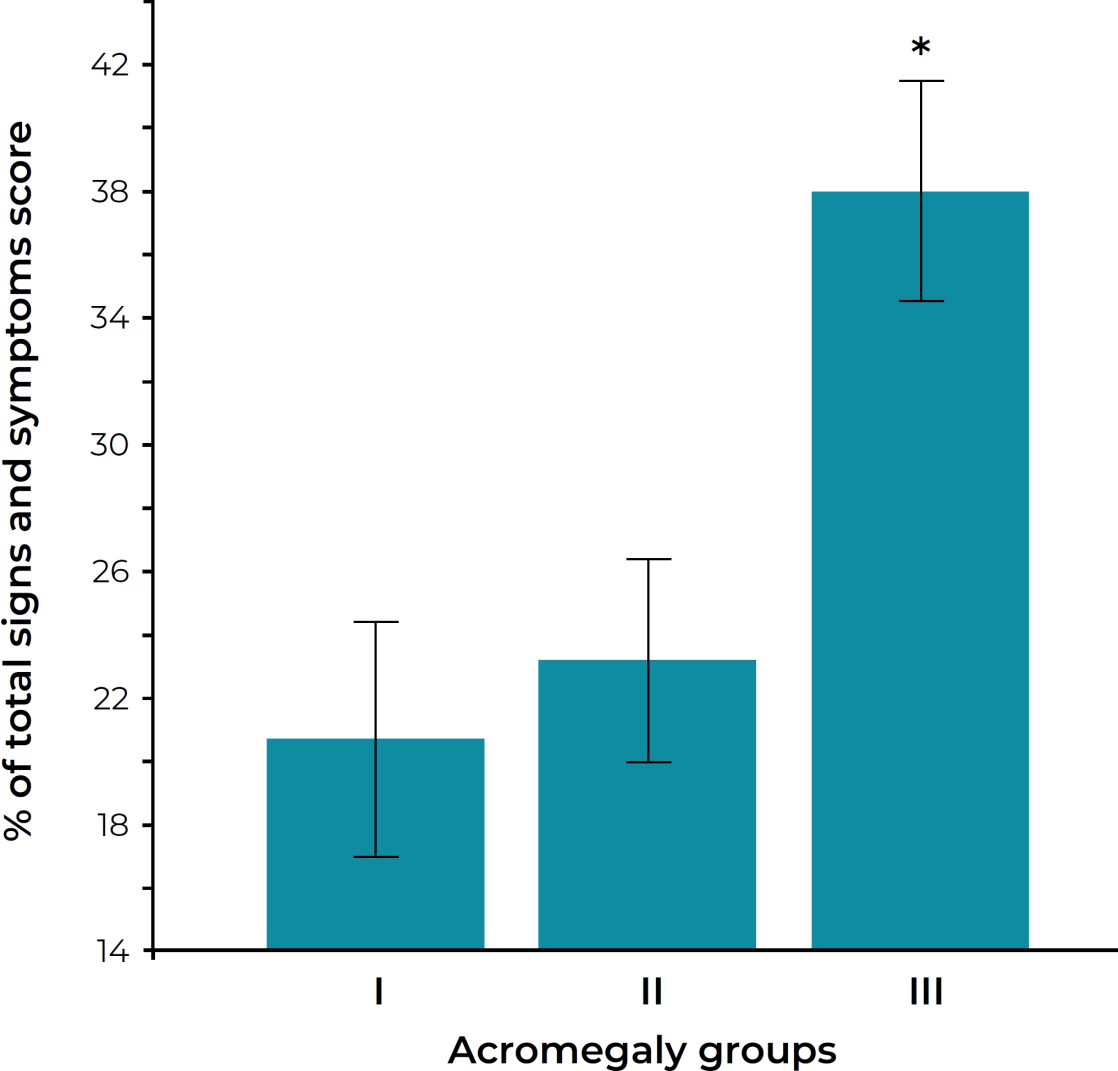
Sign and symptom score in three groups of acromegaly patients. The groups are defined as follows: group I, normal serum IGF-I levels and nadir GH after oral glucose less than 0.14 μg/liter; group II, normal IGF-I levels but nadir GH 0.14 μg/liter or more; group III, elevated serum IGF-I levels. ^*^P < 0.005 versus groups I and II. Figure reprinted with permission from Puder et al. *J Clin Endocrinol Metab* 2005;90(4):1972-1978, © The Endocrine Society (39).

Measurements of GH and IGF-I correlate with symptom burden of acromegaly. Puder et al. evaluated patients with acromegaly who underwent assessment of fasting IGF-I and fasting and post-oral glucose tolerance test GH (39). In this study, elevated IGF-I was clearly associated with excess acromegaly symptom burden (**Figure 2**) and symptom burden was not minimized unless both IGF-I and GH were completely controlled. These data support the recommendation that normal IGF-I and low GH are reasonable targets for all acromegaly patients with ongoing active symptoms of disease. However, before committing patients to combination medical regimens in order to achieve stringent biochemical control, it would be wise to clinically assess the nature of the ongoing symptoms, remembering that pre-existing permanent structural changes (e.g. joint damage from long-standing disease) may cause symptoms that are not reversible regardless of degree of disease control.

### 10.2 Longevity

One of the primary goals of medical therapy has been to restore life expectancy to normal, since it is reduced in poorly controlled acromegaly (1). All analyses of longevity have been conducted retrospectively; therefore, there are limitations on the strength of the conclusions from each of the studies. It is important to note that significant changes in assay methodology or reference ranges may have occurred during the study interval. However, there is significant agreement among the studies that life expectancy is reduced in patients who are symptomatic at the last visit or have clinical evidence of disease progression (17).

There has been a great deal of interest in whether measurement of GH or IGF-I has the best predictive value in determining reduced life expectancy. Initial studies used almost solely GH measurements; however, more recent studies have also utilized IGF-I, and several studies have compared the ability of each measurement to predict outcome. Initial studies that utilized the less sensitive GH radioimmunoassays determined that a GH value greater than 5 ng/mL was associated with increased relative risk of premature death (87, 88). More recent studies using competitive radioimmunoassays have lowered this value to 2.5 ng/mL (89). The development of more sensitive two-site monoclonal antibody sandwich assays has led to a recommendation that GH be lowered to concentrations <1.0 ng/mL; however, the validity of using this as a standard to predict reduced mortality has not been definitively demonstrated (90). Interestingly, when results were analyzed using multifactorial linear regression, thereby taking into account other variables, such as age, hypertension, duration of disease symptoms, and comorbidities, and evaluating GH as an independent predictor of mortality, a value of >5 ng/mL was needed for GH to be independently associated with increased relative risk (91). This study is consistent with others which have not found measurable increased mortality risk in the presence of mild degrees of active disease.

IGF-I measurements enable detection of subtle abnormalities of GH secretion, and, because of this, it has been hoped since their advent that they would have greater predictive value. There are studies clearly showing that IGF-I results within the reference range are associated with improvement in longevity (20, 64, 92, 93). However, other studies that utilized multifactorial linear regression have shown IGF-I is not an independent predictor of mortality risk (91, 94). A frequently cited study showed that GH >2.5 ng/mL and an abnormal IGF-I predicted an increased risk of premature mortality if univariate analysis was used; however, multifactorial analysis showed that only a GH >5.0 ng/mL independently predicted increased mortality (94). The reason for this discrepancy is not clear, but it is likely related to the proportion of patients included in the analysis who had relatively poor control of disease activity at the time of the last visit. This conclusion is supported by a recent analysis of the value of multiple GH measurements collected over a long time interval as compared to a single measurement at the last visit. In this study, the investigators utilized time-dependent exposure to high GH values and compared this to the last visit value (90). They determined that exposure to excessive GH over time was a better independent predictor of prognosis than simply using the last measured GH or IGF-I value, and concluded that the last measured value overestimated the degree of relative risk compared to time-dependent exposure. Importantly, an abnormal IGF-I was not an independent predictor of mortality (94).

**Figure 3:**
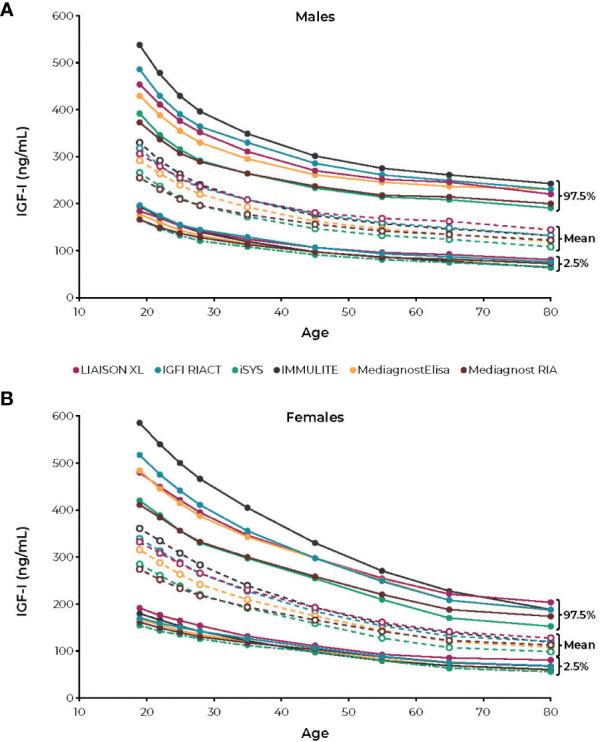
Reference intervals for **(A)** males and **(B)** females according to the age intervals of the six IGF-I immunoassays. IGF-I, insulin-like growth factor. Figure reprinted from Chanson et al; Variete Investigators (59). CC-BY-NC license, https://creativecommons.org/licenses/by-nc/4.0/.

Most of these studies took several years to complete. During this time, long-term mortality studies may have used more than one assay or assays that changed standards during the study period. These changes call into question the validity of the conclusion that IGF-I must be in the “normal” range to normalize lifespan. To highlight the limitation of such a blanket recommendation, a recent report showed that six reference labs reported very different absolute concentrations for pure IGF-I standards (**Figure 3**) (59). Changing values of the internal standard alone can account for more than 30% differences in IGF-I values (95), making it important to understand the assay being used to assess a given patient and its quantitative relationship to assays used in published mortality studies.

An additional problem has been the evolution of reference ranges during these study intervals. Prior to 2003, most labs used inadequate numbers of normal subjects to define their reference ranges. In 2003, Nichols Institute reported normative data on 3961 normal subjects that clearly showed the importance of an adequate number of subjects to properly define reference ranges by age (96). A more recent report using >15,000 normal subjects has shown that the use of an adequate sample size overcomes the need to correct reference ranges for BMI or estrogen status (58). Importantly, none of the studies that have analyzed life expectancy outcomes have used either of these assays or normative data sets exclusively.

**Figure 4:**
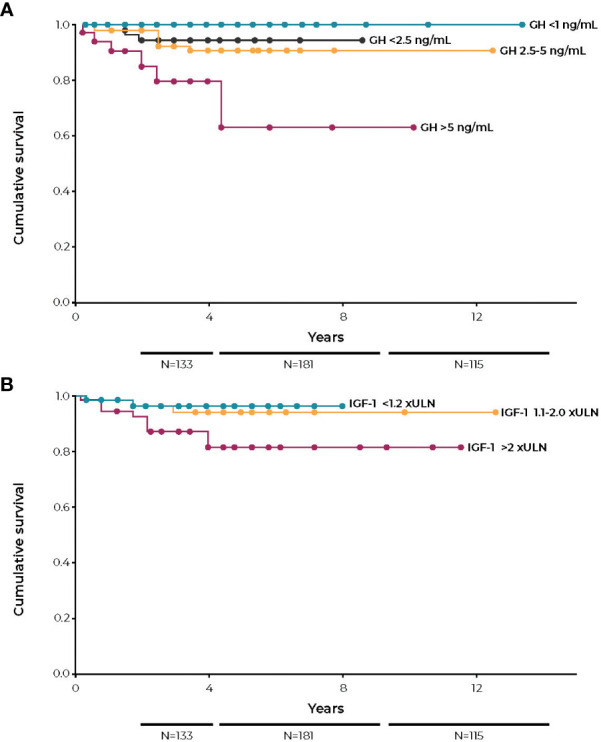
Kaplan-Meier survival curves according to last visit GH **(A)** and last visit IGF-I **(B)**. GH, growth hormone; IGF-I, insulin-like growth factor; ULN, upper limit of normal. Figures reprinted with permission from Mercado et al. *J Clin Endocrinol Metab* 2014;99(12):4438-4446, © The Endocrine Society (64).

Most studies reporting mortality outcomes based on IGF-I measurements have not analyzed patients with mild disease as a separate group. It would be of great interest to know the prognosis of patients with IGF-I values between 1.0× and 1.3× ULN as compared to patients with IGF-I values >1.3× ULN. The only study to examine this, Mercado et al, found no increased mortality risk for patients with IGF-I values <1.2× ULN. For patients with IGF-I between 1.2× to 2.0× ULN, there was a possible small increase in mortality compared to patients with IGF-I values <1.2× ULN. Only patients with IGF-I >2× ULN, however, had a marked increase in mortality (**Figure 4**) (64).

By contrast, GH analyses in some studies have subdivided patients with abnormal GH values into those with <5.0 ng/mL versus >5.0 ng/mL, finding that patients in the higher GH value group had a higher relative risk, even though GH values were abnormal in both groups. This strongly supports the conclusion that there are gradations of disease severity and that those with more severe disease carry the greater risk of premature mortality. This conclusion is also supported by comparing the relative risk in mortality in more recent outcome studies to those conducted several years ago. Recently, the control of disease activity has improved, and the relative risk of premature mortality has been reduced. (i.e. 1.5- to 1.7-fold versus >2.0-fold in older studies) (4, 89, 93, 94, 97–101). Notably, mean IGF-I and GH values in these more recent studies are significantly lower than those in older studies. This suggests that a study that analyzed outcomes in participants with lower but abnormal IGF-I and GH values would show an improved prognosis as compared to studies that included all patients with increased values in one group, regardless of their degree of biochemical control.

## 11 Translating reference ranges used in mortality studies to modern standards

The reference range for any clinical assay, including the upper limit for IGF-I, is not established based on clinical outcomes. By definition, the reference range excludes 2.5% of patients at each end of the range who are truly “normal”. In the case of IGF-I, the upper limit of the reference range used in the mortality studies published beginning in 1998 was based on measurement methods and reference ranges which are now, for all intents and purposes, obsolete (102). Nevertheless, it is important to be aware that outdated reference ranges for IGF-I are still cited (e.g. American Board of Internal Medicine test reference ranges (58, 102, 103); **Table 3**) and, therefore, it is incumbent upon the practicing physician to base the appropriate target to the assay/reference range used to measure IGF-I in the local practice environment.

**Table 3:** Reference ranges for IGF-I.

Age	Historical upper limit of reference range^*^^†^ (ng/mL)	Upper limit of reference range based on large normative database^‡^ (ng/mL, males)
18	780	494
25	492	355
30	492	282
40	360	233
50	360	205
65	290	188
80	290	172

IGF-I, insulin-like growth factor.

*From Kratz et al (99).

^†^From American Board of Internal Medicine laboratory (100).

^‡^From Bidlingmaier et al (55).

Based on large normative databases, the upper limit of modern reference ranges for IGF-I are approximately 30-50% lower than less stringent reference ranges used in previous studies (58). Endocrine consensus guidelines recommend targeting a “normal” IGF-I (e.g. within the reference range); however, this may only be appropriate as a blanket recommendation when using an older IGF-I measurement method and associated reference range. There are no mortality or long-term outcome studies that have used the recently improved assays and well-defined reference ranges exclusively. Therefore, clinicians using these assay methods and reference ranges need to consider IGF-I values as a continuum and not conclude that there is an absolute cutoff value that must be achieved. If normalization of IGF-I and GH are achieved using a modern assay with medical therapy, one can assume that medical therapy is adequate, since most of these patients will be asymptomatic (**Figure 2**) (39), but if IGF-I is between 1.0× and 1.3× ULN, the decision to further intensify therapy should be individualized. This decision, which may involve the institution of a complex medical regimen, should take into account the presence of residual symptoms or comorbidities, medication risks, impact of medication burden on quality of life, as well as the substantial financial costs.

## 12 Conclusions

In summary, measurement of both GH and IGF-I has undergone significant evolution during the past 40 years. During this time there have been major improvements in measurement of both hormones, and the sensitivity and specificity of both assays have been markedly improved. Furthermore, experience with utilization of these techniques has become more widespread and, therefore, the strength of the conclusions that can be drawn from these measurements has advanced. Finally, adoption of large normative reference ranges that take into account age, BMI, estrogen status, and the presence of other confounding variables has improved. Although these changes have made current assay results much more reliable, the upper limit of the reference range has proven to be lower than in historical assays, making comparison of studies across these decades more difficult.

For patients not cured by transsphenoidal surgery or radiation therapy, achieving IGF-I in the reference range using first-line pharmacotherapy (somatostatin agonists; or cabergoline for mild disease) is desirable. When first-line pharmacotherapy has been optimized and IGF-I remains between 1.0× and 1.3x ULN in an assay that uses a modern reference range (58), we recommend that the total spectrum of information available be utilized to make the decision as to whether additional therapy should be undertaken. This would include the presence of symptoms, progression of comorbidities, GH values, degree of improvement in IGF-I on prior therapy, medication burden, and financial considerations.

## Author contributions

DC contributed to conceptualization, drafted the manuscript, and critically reviewed and revised the manuscript. MB contributed to the conceptualization and critically reviewed and revised the manuscript. All authors contributed to the article and approved the submitted version.
